# Supplementary material for: Holographic tomographic volumetric additive manufacturing
Source: Nat Commun. 2025 Feb 11;16:1551. doi: 10.1038/s41467-025-56852-4 (PMC11814129; doi:10.1038/s41467-025-56852-4)
Supplement: Supplementary file 1 — Supplementary Information [file 41467_2025_56852_MOESM1_ESM.pdf]

Supplementary Information

Holographic Tomographic Volumetric Additive Manufacturing

Maria I. Álvarez-Castaño[1], Andreas Gejl Madsen[2], Jorge Madrid-Wolff[1,3], Viola Sgarminato[1], Antoine Boniface[1,4], Jesper Glückstad[2], Christophe Moser[1*]*

[1]*Laboratory of Applied Photonics Devices, School of Engineering, Ecole Polytechnique Fédérale de Lausanne, CH-1015, Lausanne, Switzerland*
[2]*SDU Centre for Photonics Engineering, University of Southern Denmark, DK-5230 Odense M, Denmark*
[3] *current address: Readily3D, Renens, Switzerland*
[4] *current address: AMS Osram, Martigny, Switzerland*

**Corresponding authors: Maria I. Álvarez-Castaño, maria.alvarezcastano@epfl.ch; Christophe Moser, christophe.moser@epfl.ch*

Supplementary Note 1. Experimental setup

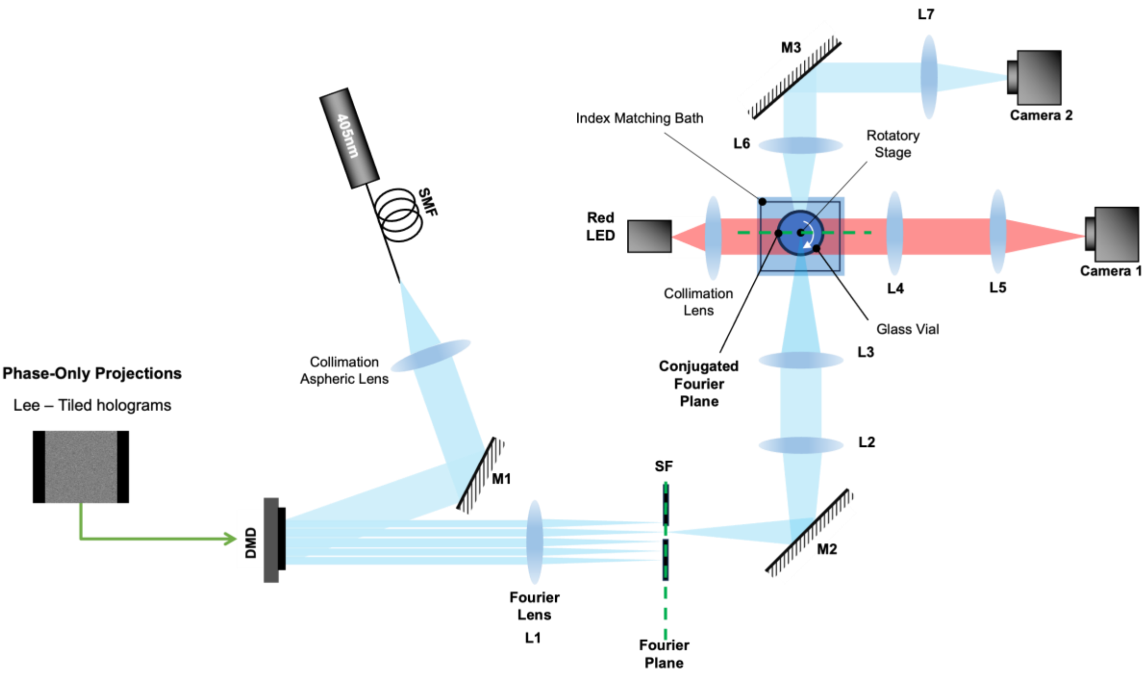

Supplementary Figure 1. Experimental setup of the Tomographic Volumetric 3D printing by phase encoding (HoloVAM). A Fourier lens L1 reconstructs the hologram at the Fourier plane, which is then imaged into the rotatory resin container. Using lenses L2 and L3 we conjugate the Fourier plane (green dashed line illustrates the conjugated planes).

Supplementary Note 2. Hologram test: Speckle noise

Here, we experimentally compare the intensity reconstruction generated by two spatial light modulators: a liquid crystal SLM (LC-SLM) which is a phase SLM and a DMD which is a binary

amplitude device. For the latter, the Lee Hologram method converts binary amplitude modulation to phase modulation. We generated two CGHs, corresponding the letter "A" and "E" respectively using the GS algorithm. We used two tiles to generate the CGHs. For both SLMs, speckles can still be seen (Supplementary Figure 2, a,c). Speckles are reduced when six CGHs are time multiplexed as expected. Each of the 6 CGHs was constructed by adding a random phase to each tile. The time multiplexed reconstructions showed less speckle noise.

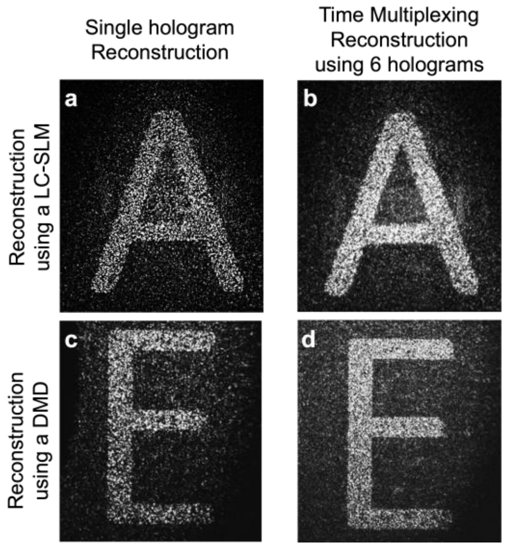

Supplementary Figure 2. Reconstruction comparison between phase and amplitude SLMs. a experimental intensity reconstruction of letter "A" by the LC phase SLM (experimental setup in Supplementary Figure 3). **b** Averaged reconstruction of 6 time multiplexed CGHs of the letter "A". **c** experimental intensity reconstruction of letter "E" by a DMD **d** Averaged reconstruction of 6 time multiplexed CGHs of the letter "E".

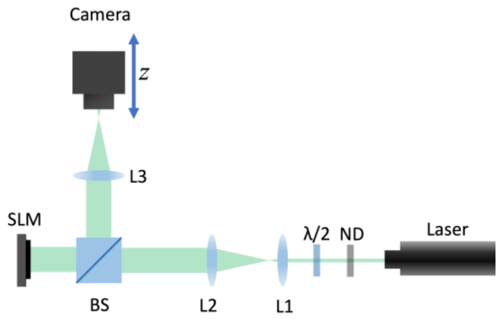

Supplementary Figure 3. Experimental setup to test CGHs using LC-SLM. A CW laser at 532 nm was used as light source. Lenses $L1$ and $L2$ were used to expand the beam by a factor of 5. A liquid crystal SLM (Holoeye PLUTO NIR II) was used to display the CGH. A $\lambda/2$ waveplate is used to generate the appropriate linear polarization on the SLM . Normal incidence on the SLM was chosen to avoid additional aberrations in the reconstruction. The reconstructed images were captured using a monochromatic CMOS camera with a pixel size of 2.2 μm.

Supplementary Note 2.1. Hologram test using DMD

Here we reconstruct the letter "A" tiled with a different amount of tiles on a DMD (1024 X 768, pixels with size 13.76 μm). We performed the experiment using the experimental setup in Supplementary Figure 1. Results are shown in the figure below. Compared with the reconstructions of CGH without tiling (Supplementary Figure 2), we observe that tiling reduce the level of speckles.

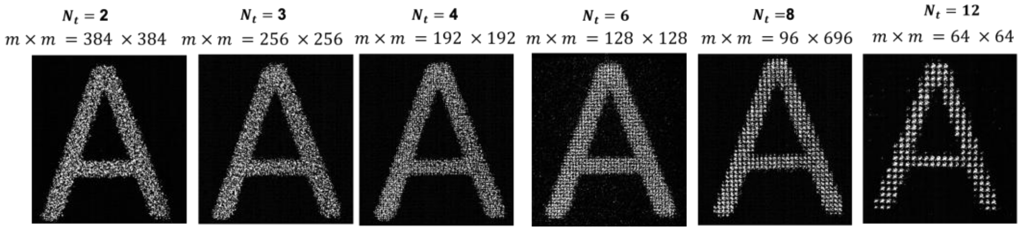

Supplementary Figure 3.1. Experimental reconstruction of tiled hologram of the "A" letter for a different number of tiles using a DMD**.**

Supplementary Note 3. Light efficiency measurements

The patterns in Supplementary Figure 4a can be generated using phase coding instead of amplitude coding. In the former, all pixels in the modulator plane contributes to each pixel in the image plane. To measure the efficiency of the DMD SLM using phase holograms coded with the Lee method, we use the experimental setup shown in Supplementary Figure 1. First, we measured the power in the focal of the printing plane when all the pixels of the DMD are "ON". We obtained P_{in} = 4.61 mW. For amplitude coding, the pattern in Supplementary Figure 4 a is loaded on the DMD. We measured the projected power $P_{out}^{incoherent} = 0.016\, mW$, providing an efficiency of the incoherent patterns of $\eta_{incoherent} = 0.34\%$. For a coherent pattern instead, we measured the power in the conjugate plane of the Fourier when all the pixels of the DMD are "ON". We obtained P_{in} = 6.46 mW. Then, the CGH corresponding to the phase pattern that allows the reconstruction in Fig. 4b is loaded on the DMD, the power in the conjugate plane of Fourier plane after the 4F is measured as $P_{out}^{coherent} = 0.627\, mW$, giving an efficiency of $\eta_{coherent} = 9.71$ %. The light efficiency here could be further increased

if the full size of the DMD was used. The CGH has a size of 768 x 768 pixels due to the constraints of the PSF shaping.

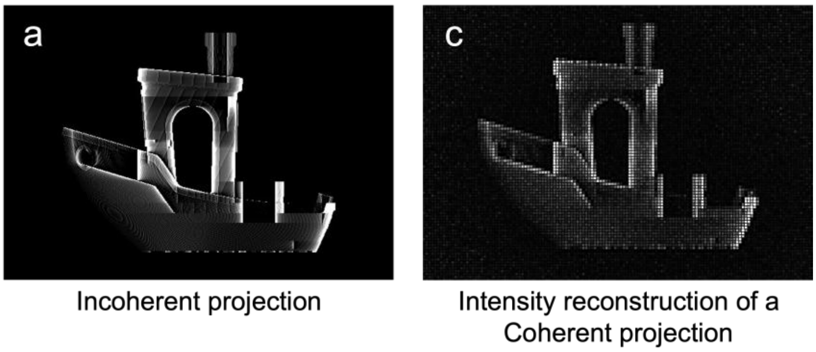

Supplementary Figure 4. a Incoherent projection. Amplitude coded pattern corresponding to the projection angle $\theta = 0°$. **b** Coherent projection. Intensity reconstruction of coherent projections generated using the amplitude pattern in (a) as target intensity.

Supplementary Note 4. Sampling Rate and Resolution

Since the system is diffraction-limited, the voxel resolution is given by $\propto \lambda/NA$. The numerical aperture, NA, is a function of the sub-hologram size, taken as the aperture size, that changes with the number of tiles, and the magnification of the 4*f* system. The computation considers a DMD matrix of 768 X 768 pixels with a pixel size of $13.7\ \mu m$, and a wavelength $\lambda = 405\ nm$. For a magnification of 1.3x and number of tiles $1 < N_t < 5$, the spatial resolution is $1.5\ \mu m < \lambda/NA < 9.2\ \mu m$ respectively (Supplementary Figure 5a). As an example, 4 tiles ($N_t = 4$) yields a voxel resolution of $\sim 6.2\ \mu m$. The computation considers a full matrix of 768 X 768 pixels with a pixel size of $13.7\ \mu m$, and $\lambda = 405\ nm$.

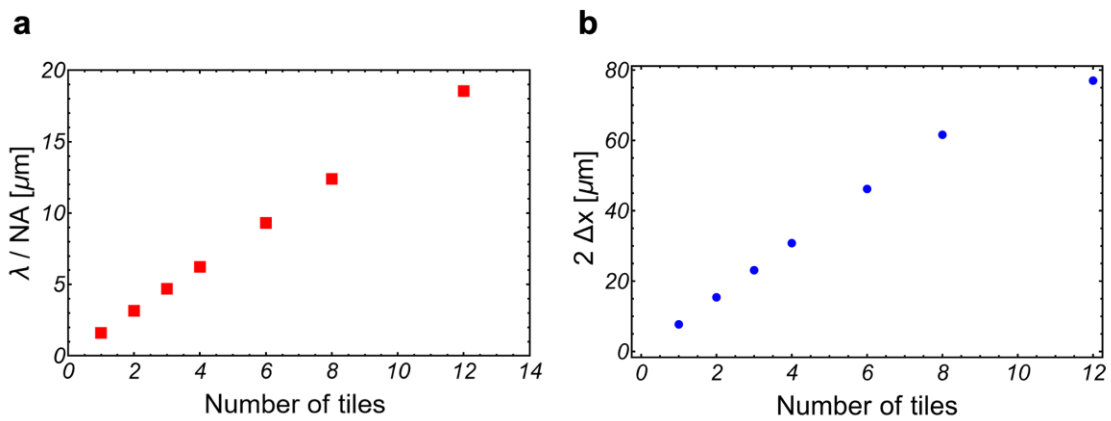

Supplementary Figure 5. a Voxel resolution as a function of the number of tiled holograms. **b** Sampling rate calculation for different tiled holograms

Supplementary Note 5. Tomographic projections

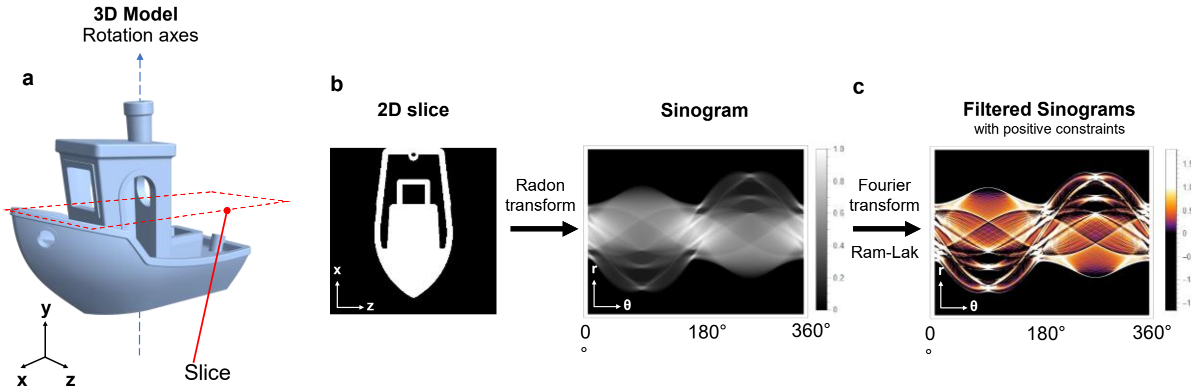

Supplementary Figure 6. Pipeline for computing the amplitude projections. a 3D target object. Benchy boat (Copyright CC) render produced with Wolfram Mathematica® 13.1. The dashed lines represent a plane slicing the object perpendicular to the direction of rotation. **b** A 2D slice from the 3D object. **c** Sinogram after applying the Radon Transform. **d** Filtered back projections after applying a Ram-Lak filter and setting negative values to zero.

Supplementary Note 6. Gerchberg-Saxton (GS) iterative method

Computer-generated holograms (CGH) were synthesized using the Gerchberg-Saxton (GS) algorithm [1–3] to generate the desired intensity pattern in the sample plane by modulating the phase of the input wavefront of a coherent light beam. Here, the tomographic projections previously computed using the filtered back projections were used as target images. To obtain a 2D phase mask (CGH), the algorithm performs *m* iterations by simulating forward and

backward propagation of the light field from the sample plane to the Fourier plane via a direct and inverse fast Fourier Transform (Supplementary Figure 7.a-b). A new CGH is computed for every projection.

Supplementary Figure 7.c illustrates the block diagram of the traditional GS algorithm. In the first iteration, an initial random phase $\Phi_0(x, y)$ is generated. Then, this phase is applied to the target amplitude at the image plane $\sqrt{I_T} \times \exp[j \times \Phi_0].]$. The field is inverse Fourier transformed to the hologram plane, where the amplitude u is discarded and replaced by the amplitude of the illumination source $\sqrt{I_s} \times \exp[j \times \varphi]$. The new complex field is Fourier transformed again to the image plane. The resulting amplitude U is replaced by the target intensity and the newly computed phase $\Phi(x, y)$ is kept. After performing m iterations, the final phase $\Phi_m(x, y)$ is used as the phase CGH [1,3].

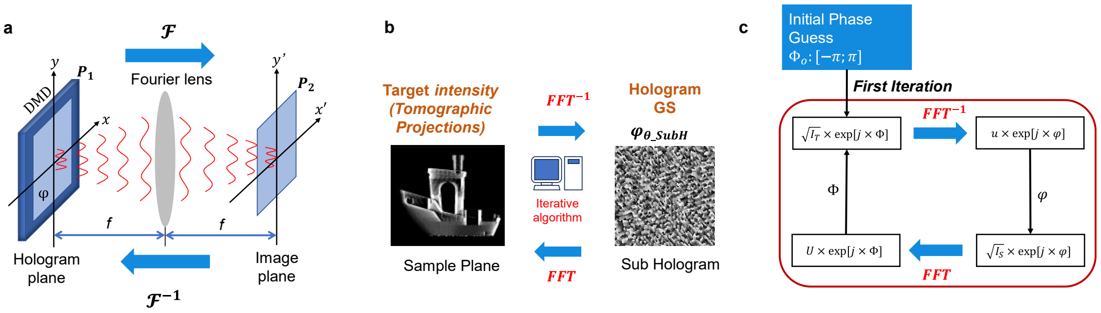

Supplementary Figure 7. a Schematic diagram to illustrate the forward and backward propagation of the light in a GS algorithm. **b** Block diagram of the forward and backward propagation of the GS algorithm using Tomographic projections as target intensities. **c** Block diagram of a traditional GS algorithm.

Supplementary Note 6.1. Mean Squared Error (MSE) and Structural Similarity Index (SSMI)

We use two different metrics to quantify the CGH projection error: the Mean squared error (MSE) and Structural similarity index (SSIM) offer complementary insights into the projection fidelity when speckle noise is present. To cover a projection depth of 4mm, we evaluate the error as a function of an increasing number of projecting planes from 4 to 34. With 34 projections, a CGH is optimized every 117.6 µm whereas for 4 projections, a CGH is optimized

every 1 mm. We observe that the uniformity of the error across the propagation depth increases with the number of CGHs but that the fidelity as measured by SSIM and MSE decreases with increasing CGHs. A tradeoff exists between error uniformity across the build volume and fidelity. We have selected 6-8 CGHs (corresponding to a sampling of z 500 μm – 666μm) which is a good compromise between fidelity and error uniformity.

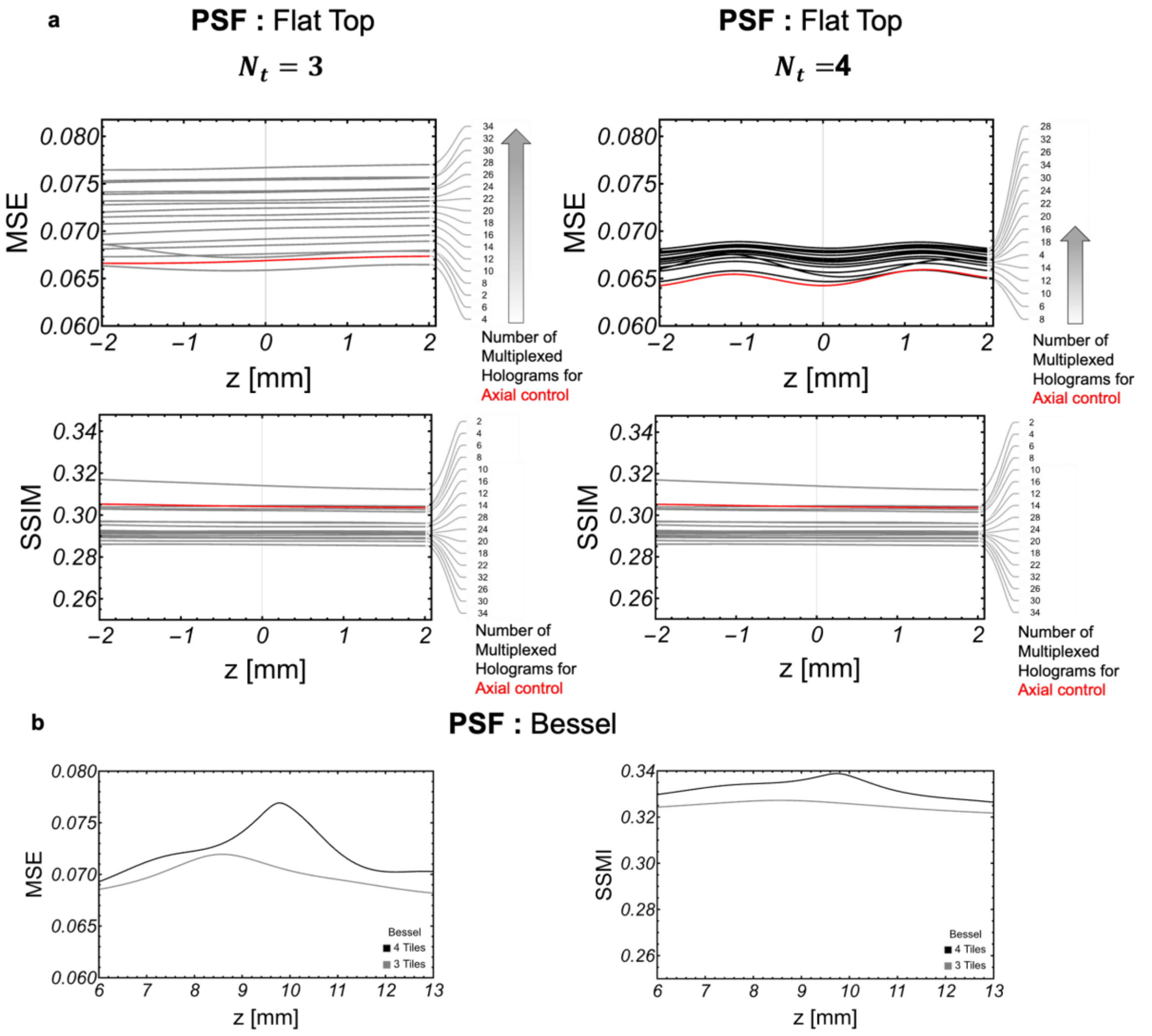

Supplementary Figure 7.1. **a** Mean square error (MSE) and Structural similarity index (SSIM) of the simulated intensity reconstruction of the tiled holograms as a function of depth z in the vial compared with the target amplitude tomographic patterns for a number of tiles $N_t = 3$ (left) and $N_t = 4$ (right) using a Flat-top PSF. **b** MSE and SSIM measured from simulated intensity reconstructions of tiled holograms using Bessel PSF, compared with the amplitude tomographic pattern as a target. The simulation was made for the projected angle $\theta = 0°$.

Supplementary Note 7. HoloTile formalism

We assume a *phase-only* pattern displayed on a phase SLM (or indirectly from a binary amplitude DMD using Lee holograms [7,8]). The spatial light field reflected off the SLM can be described as:

$$a = b(x,y)\mathrm{rect}\left(\frac{x}{X_{SLM}},\frac{y}{Y_{SLM}}\right)\exp\big(i\varphi_{PSF}(x,y)\big)\big[\exp\big(ih(x,y)\big)\otimes \mathrm{comb}(x\Delta X, y\Delta Y)\big] \quad (1)$$

where $b(x,y)$ is the input beam shape, $\mathrm{rect}\left(\frac{x}{X_{SLM}},\frac{y}{Y_{SLM}}\right)$ describes the rectangular aperture of the SLM, $h(x,y)$ is the calculated CGH to be tiled, and $\varphi_{PSF}(x,y)$ is an independent PSF-shaping hologram phase. The tiled hologram $\varphi_{tile}(x,y)$ is conveniently described by the convolution $[\exp\big(ih(x,y)\big)\otimes \mathrm{comb}(x\Delta X, y\Delta Y)]$.

The resulting complex field reconstruction at the optical Fourier plane of the 2*f*-setup is then:

$$\begin{aligned}\tilde{O} = \frac{X_{SLM}Y_{SLM}}{\Delta X\Delta Y}\mathrm{sinc}\big(X_{SLM}f_x\,, Y_{SLM}f_y\big)\otimes \Im\big(b(x,y)\big)\otimes \Im\big(\exp(i\varphi_{PSF}(x,y)\,)\big)\\ \otimes\left[\Im\left(\exp\big(ih(x,y)\big)\right)\mathrm{comb}\left(\frac{f_x}{\Delta X},\frac{f_y}{\Delta Y}\right)\right]\end{aligned} \quad (2)$$

Assuming a plane wave input beam shape, we have $\Im\big(b(x,y)\big)\to\delta(0{,}0)$ and therefore equation (2) simplifies to:

$$\begin{aligned}\tilde{O}_{\rightrightarrows} = \frac{X_{SLM}Y_{SLM}}{\Delta X\Delta Y}\mathrm{sinc}\big(X_{SLM}f_x\,, Y_{SLM}f_y\big)\otimes \Im\big(\exp(i\varphi_{PSF}(x,y)\,)\big)\\ \otimes\left[\Im\left(\exp\big(ih(x,y)\big)\right)\mathrm{comb}\left(\frac{f_x}{\Delta X},\frac{f_y}{\Delta Y}\right)\right]\end{aligned} \quad (3)$$

We can now better see the interplay between the HoloTile reconstructed output terms. The optical Fourier reconstructed output term arising from the tiled hologram $h(x,y)$ is given by:

$$\tilde{O}_H = \Im\left(\exp\big(ih(x,y)\big)\right)\text{comb}\left(\frac{f_x}{\Delta X},\frac{f_y}{\Delta Y}\right) \tag{4}$$

which basically describes a *sampled* reconstruction of the Fourier transformed hologram phase. The output term arising from the independent PSF-shaping hologram phase is given by:

$$\tilde{O}_P = \Im\left(\exp\big(i\varphi_{PSF}(x,y)\big)\right) \tag{5}$$

By ignoring the diffraction impact from the input SLM/DMD aperture truncation – corresponding to approximating the term $\text{sinc}\big(X_{SLM}f_x\,,Y_{SLM}f_y\big) \rightarrow \partial(0{,}0)$ with an output centered Kronecker delta-function for increasing aperture sizes - we finally have the following simple expression for the HoloTile Light Engine reconstructed complex output field:

$$\tilde{O}_{\rightrightarrows} \propto\ \tilde{O}_P \otimes \tilde{O}_H \tag{6}$$

Let us first consider the optical Fourier reconstructed output term, $\tilde{O}_H$, arising from the tiled hologram *h* displayed at the input spatial light modulator or DMD:

$$\tilde{O}_H = \Im\left(\exp\big(ih(x,y)\big)\right)\text{comb}\left(\frac{f_x}{\Delta X},\frac{f_y}{\Delta Y}\right) \tag{7}$$

This term can be illustrated as a discretized delta-function and amplitude weighted version of the "continuous" holographic reconstruction $\Im\left(\exp\big(ih(x,y)\big)\right)$.

the PSF-shaping hologram phase $\tilde{O}_P$ is 2D-convolved onto the delta-function discretized and amplitude weighted holographic reconstruction

$$\tilde{O}_P = \Im\left(\exp\big(i\varphi_{PSF}(x,y)\big)\right) \tag{8}$$

The resulting effect can be illustrated by superposing $\Im\left(\exp\big(i\varphi_{PSF}(x,y)\big)\right)$ around each amplitude weighted delta-function discretization generated by o_H. The same applies depth-wise [6] along the optical axis *z* so that we can also write:

$$\tilde{O}_{\rightrightarrows}\big(f_x,f_y,\Delta z,t\big) \propto \widetilde{O_P}\big(f_x,f_y,\Delta z,t\big) \otimes \tilde{O}_H\big(f_x,f_y,t\big) \tag{9}$$

Supplementary Note 7.1. Point spread function modification

the PSF phase $\varphi_{PSF}(x,y)$ that, for example, converts a single-mode Gaussian beam into flat-top reconstructed voxels is computed using Supplementary Equations (10 and 11) [9,10].

$$\varphi_{PSF}(x,y) = \left[\beta_x \varphi_x(x) + \beta_y \varphi_y(y)\right] \tag{10}$$

$$\beta = \frac{2\sqrt{2\pi} r_0 y_0}{f\lambda} \tag{11}$$

where λ is the wavelength, r_0 is the radius at $1/e^2$ point of the input Gaussian beam, y_0 is half-width of the desired dimension in the reconstruction plane, and f is the focal length of the Fourier lens [9–11]. The $\varphi_{PSF}(x,y)$ is dependent on β and the phase element $\varphi(\xi)$. It is necessary to calculate β and $\varphi(\xi)$ for each dimension.

Where, $\varphi(\xi) = \frac{\sqrt{\pi}}{2}\xi\,\mathrm{erf}(\xi) + \frac{1}{2}\exp(-\xi^2) - \frac{1}{2}$, and $\xi = \frac{\sqrt{2}\cdot x}{r_0}$ or $\xi = \frac{\sqrt{2}\cdot y}{r_0}$.

The relationship between the Fourier plane and the DMD plane (aperture) is given by the relation [5,12]:

$$\Delta x = \frac{\lambda f}{w_x} \tag{12}$$

where λ is the wavelength of the light, f is the focal length of the Fourier lens, w_x is the half-width of the aperture, and Δx is the half width of the period of the interference pattern at the Fourier plane. Considering the physical parameters related to the DMD and sub-holograms we can rewrite the equation as:

$$\Delta x = \frac{N_t \lambda f}{2L \cdot \ell_{px}} \tag{13}$$

Where ℓ_{px} is the pixel size. From equation (13), it is possible to see that for each tile there is a modification of the sampling function which is related to the half-width of the desired flat-top

dimension in the Fourier plane, as the number of tiles modifies the separation between the image points.

After adding a phase function to provide a flat-top reconstructed pixel, the final phase of the tiled CGH encoding by HoloTile is:

$$\varphi_{Tiled} = \varphi_{tile} + \varphi_{PSF} \tag{14}$$

Supplementary Note 8. Phase for Axial Control and non-diffractive beams

Supplementary Note 8.1. Fresnel Lenses

Axial control can be achieved by adding a Diffractive Optical Element (DOE), in this case, a Fresnel lens, with the designed hologram before the encoding to a binary hologram. The phase lens [13,14] is expressed as:

$$\varphi_{Len}(x, y, z) = \frac{2\pi z}{\lambda f^2}(x^2 + y^2) \tag{15}$$

where z is the desired shift of the reconstruction plane relative to the focal length f of the Fourier lens. Adding the Fresnel lens phase $\varphi_{Len}(x, y, z_n)$ to a tiled phase $\varphi_{Tiled}(x, y)$ moves the reconstruction plane of the tiled hologram a distance z_n around the focal plane. A stack of N holograms per projection angle were used to spread the tomographic projections with a controllable divergence over the propagation axes, each phase is calculated from Supplementary Equation (16).

$$\varphi_\theta^N(x, y, z_n) = \varphi_{Tiled}(x, y) + \varphi_{Len}^N(x, y, z_n) \tag{16}$$

Supplementary Note 8.2. Bessel Beams

Alternatively, to provide a collimated beam to fulfill the assumption of the Radon transform, we can use a Bessel beam (also called “non-diffractive” beam) as an alternative to lengthen the projection distance on the building volume.

There are many ways to generate a Bessel beam. One way consists of sending a Gaussian beam through an axicon which is easily produced using a spatial light modulator. The phase function of a single axicon, axially center is expressed as [15]:

$$\varphi_{Bessel}(x,y) = -\frac{2\pi}{\lambda}(n-1)\alpha\sqrt{x^2+y^2} \qquad (17)$$

where λ is the wavelength of the incident beam, n is the refractive index of the axicon, and α is the bottom angle of the axicon. The maximum base angle of the Axicon is $\alpha_{max} \leq \frac{\lambda}{2(n-1)d}$, where d is the pixel pitch of the SLM. Supplementary Figure 8a illustrates how the axicon phase creates a focal line (z_{max}) over the optical axis thanks to interference. Supplementary Fig. 8b shows a typical axicon phase "en face". Supplementary Fig. 8c shows the axial intensity generated by an axicon with the following parameters: refractive index n = 1.52, base angle $\alpha = 0.02°$, wavelength $\lambda = 405\ nm$, hologram size $768\ \times 768\ pixels$, and pixel pitch of $13.7\ \mu m$. Different, base angle and refractive index generate a different axial intensity distribution. The parameters above were set to have an extended focus over the printed volume (2.5 mm).

Rather than a stack of N holograms per projection used to synthetically control the beam collimation, using an axicon to control the divergence of the beam, only one hologram per angle is used. Each phase is calculated as:

$$\varphi_{\theta}(x,y,z) = \varphi_{Tiled}(x,y) + \varphi_{Bessel}(x,y) \qquad (18)$$

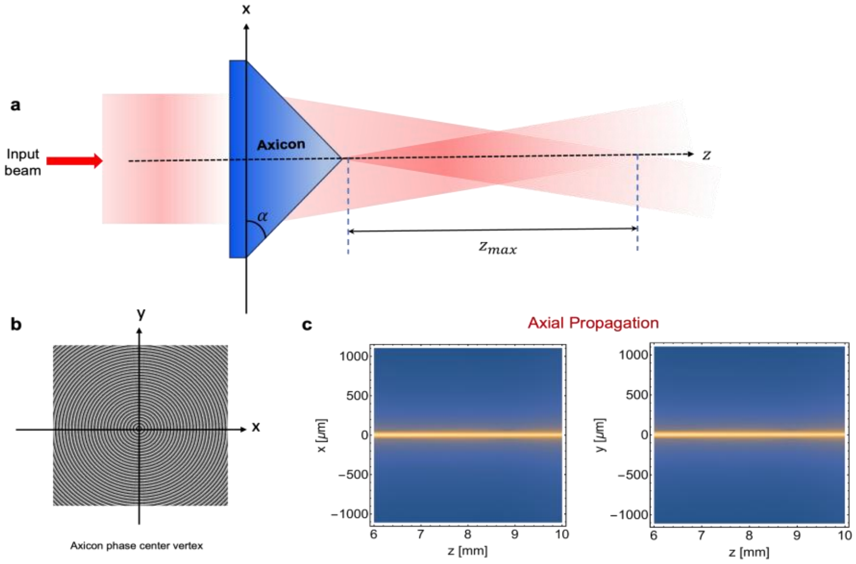

Supplementary Figure 8. Bessel Beam generation. a Schematic of the principle of generation a Bessel Beam with an axicon**. b** Phase hologram for generating the Bessel Beam added onto the SLM. The white color corresponds to a zero phase value and black color corresponds to a phase of 2π rad. **c** Axial intensity distribution of Bessel beam generated with an Axicon.

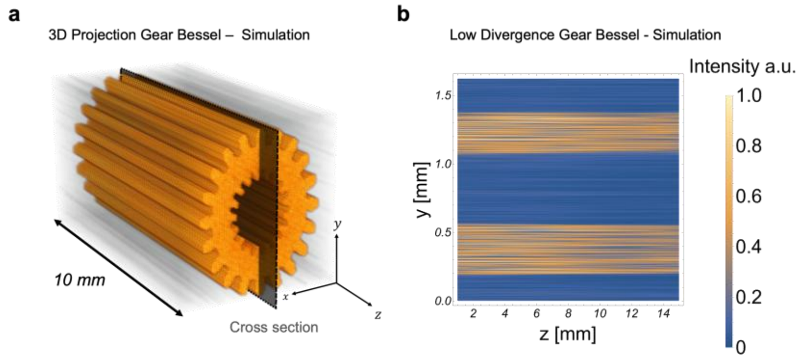

Supplementary Figure 8.1. a) 3D simulation of the cumulated light dose for one tiled hologram using a Bessel beam. **b**), axial cross-section of the cumulative dose

Supplementary Note 8.3. Optical Vortex

Optical Vortices (OV) are optical fields characterized by a phase singularity, where the phase of the light wave is undefined, and where the intensity is zero at the vortex center. These wavefronts are characterized by an integer number ℓ, called the topological charge of the vortex, which indicates the number of 2π phase changes around the vortex. Thus, ℓ

represents the speed of the phase rotation around the singularity and is related to the quantization of the Angular Momentum (AM).

The phase profile of an optical vortex can be represented in polar coordinates as

$$\psi(\rho, \varphi) = e^{i\ell\varphi} \tag{19}$$

where ℓ is the topological charge of the vortex and governs the total phase shift as the angle φ changes from 0 to 2π rad

$$\varphi_{vortex} = \ell Tan^{-1}\left(\frac{y}{x}\right) \tag{20}$$

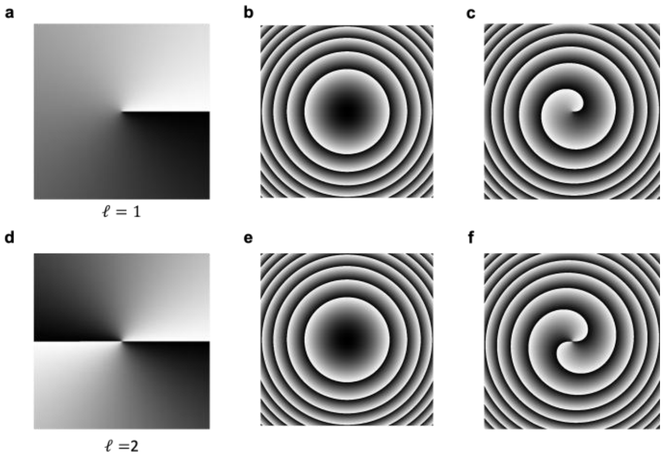

Supplementary Figure 8.2. Vortex phase masks. The white color corresponds to a zero phase value and black color corresponds to a phase of 2π rad. Phase masks **a** and **d** are plots of vortex of charge ℓ=1, and $\ell = 2$, respectively. **b** and **e** are the phase of Fresnel Lenses, calculated from Supplementary Equation (15). **c** and **f** are plots of helical phase plates created by adding the vortex phase and the lens phase.

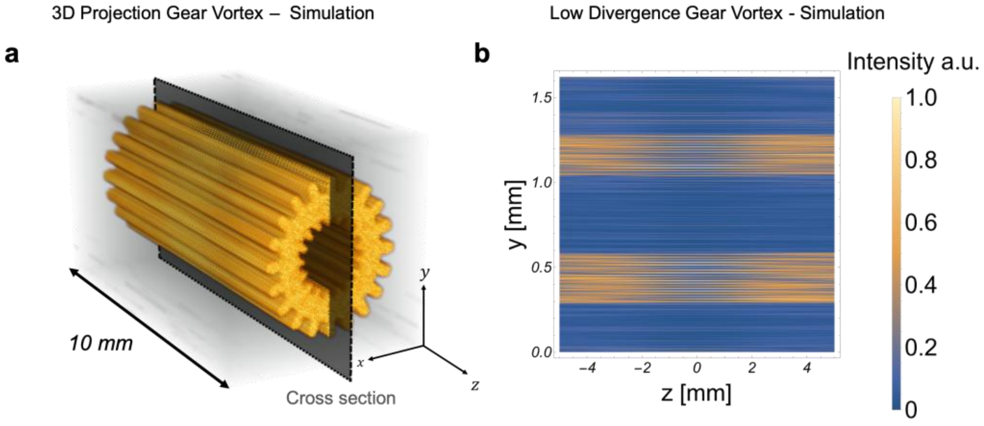

Supplementary Figure 8.3 a Left, accumulated light dose for one tiled hologram using an optical vortex projecting a gear on 10 mm distance. **b** axial cross-section of the cumulative dose

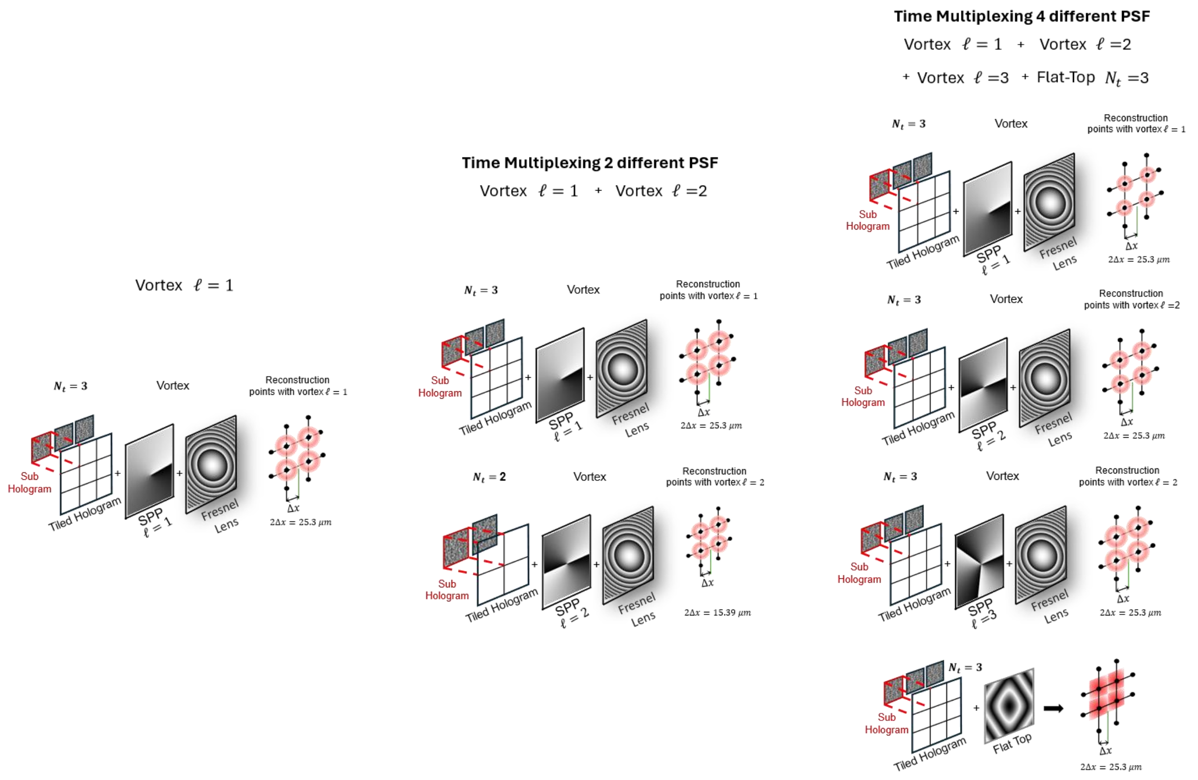

Supplementary Figure. 8.4. PSF multiplexing to reduce striation. (Left) projections using sub-holograms tiled $\mathrm{N_t} = 3$, convolved with a helical phase plate with charge $\ell = 1$. (Middle) time multiplexing different PSFs: top, $\mathrm{N_t} = 3$ with a helical phase plate with charge $\ell = 1$ and bottom $\mathrm{N_t} = 2$ with a helical phase plate with charge $\ell = 2$. (Right) time multiplexing four different PSFs.

Supplementary Note 9. Lee Hologram Method

Binary-amplitude holography enabled fast wavefront control using a DMD to display phase-encoded tomographic projections. Lee Holograms is a simple technique to modulate the amplitude $A(x, y)$ and phase $\varphi(x, y)$ of a target optical field. The binary Lee hologram hologram is computed according the following relation, Supplementary Equation (21) [7,8]:

$$H(x, y) = \frac{1}{2} + \frac{1}{2} sgn[\cos[2\pi\nu_0 x + \varphi(x, y)] - \cos[\pi\omega(x, y)]] \qquad (21)$$

Here the phase is encoded using a linear carrier in x with frequency ν_0. sgn is the sign function, and $\omega(x, y)$ is a function related with the amplitude of the target optical field as follows:

$$\omega(x,y) = \frac{1}{\pi}\arcsin\left[A(x,y)\right] \quad (22)$$

The linear carrier with frequency $\nu_0 = 1/x_0$, separates the diffraction orders in the Fourier plane. Supplementary Equation (23) is simplified for a phase-only hologram with a uniform and unitary amplitude.

Each phase set φ_θ^N is encoded in a binary Lee Hologram. Where the Lee hologram sequences displayed on the DMD are given by:

$$H_\theta^N(x,y,z_n) = \frac{1}{2} + \frac{1}{2}sgn[\cos\left[\frac{2\pi x}{x_0} + \varphi_\theta^N(x,y,z_n)\right]] \quad (23)$$

Supplementary Note 10. Wobbling correction

As previously described, holographic VAM is based on reverse tomography, where a glass resin container is rotating. The sample holder is mounted on a rotatory stage (see Supplementary Figure. 9.1a. This part consists of a clamp that holds the glass container and it is different for different resin container diameters. Wobbling occurs each time this clamp is removed and adjusted to the mandrill thread, The wobble of the resin container can cause a loss of resolution and fidelity in the printed part. A wobble correction was performed using an alignment tip, Supplementary Figure.9.1 a (right). The tip is used to align the optical axes with the rotation axes.

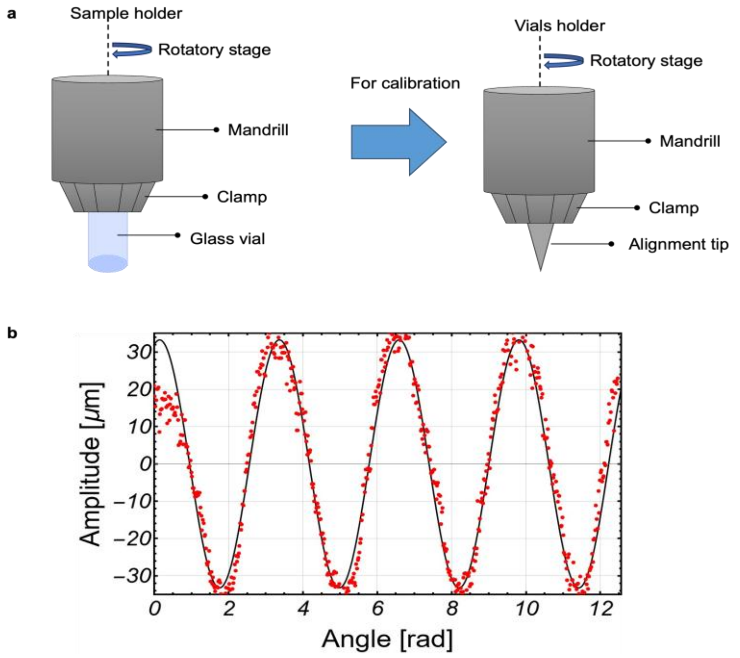

Supplementary Figure. 9.1. Wobbling measurement. a Illustration of the glass resin container holder used in the printer (left). The sample holder is mounted on the rotatory stage. (right) illustration of the calibration tip used to measure wobbling. **b** measurement of the wobbling and fitting curve in red.

Using the inspection camera 2 in our setup (Supplementary Figure 1), a sequence of images of the calibration tip is recorded every 40 ms as the rotatory stage moves at constant velocity. A DAQ National Instrument card is used to synchronize the recording system. Data analysis of the tip centroid motion shows a wobble (Supplementary Figure 9.1). A curve fit of the data points allows us to obtain the wobble. Then the wobble is corrected by adding a linear phase φ on the CGH.

$$\varphi(y) = 2\pi \cdot (y/\Lambda_y) \tag{24}$$

Where the period $\Lambda_y$is extracted from the wobble measurement as follows:

From Supplementary Figure 9.2 a and b we can relate the tilt angle and the deviation angle as $Tan(\theta) = d/f$, and $Tan(\theta) = \lambda/p \cdot \Lambda_y$. Then the wobble motion d is related to the linear period as:

$$d = \frac{\lambda \cdot f}{p \cdot \Lambda_y} \tag{25}$$

Where λ is the wavelength, f is the focal length of the Fourier lens and p the pixel pitch of the DMD.

By mapping each amplitude of the data fitted, a different period Λ_y is obtained for each *d*.

An example of how the linear phase is added to the tiled hologram is shown in Supplementary Figure 9.2c.

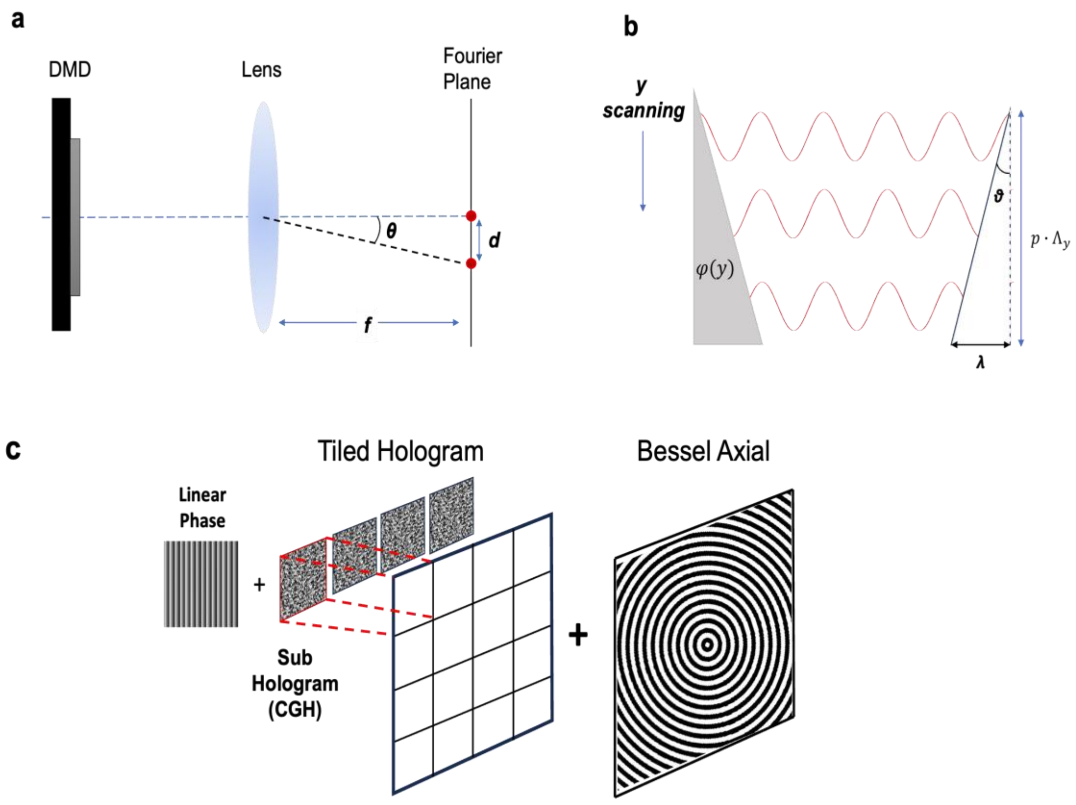

Supplementary Figure 9.2. a Illustrates the relation of a linear shift *d* in the Fourier plane of a lens with focus *f* when a linear phase is displayed on the DMD. **b** Illustration of the tilt θ produces for a linear phase using the prism equation**. c** Illustrates the linear phase addition to the sub-hologram for wobbling correction.

Supplementary Note 11. Comparison of HoloVAM to current volumetric additive manufacturing technologies

Supplementary Table 1. Comparison of HoloVAM to current volumetric additive manufacturing technologies

Supplementary Table 1. Comparison table current volumetric additive manufacturing technologies

REFERENCE WORK	TECHNOLOGY	LIGHT SOURCE	NUMBER OF BEAMS SIMULTANEOUSLY	GLOBAL TRANSFORMATION	DIGITAL AXIAL CONTROL PROJECTION	MODULATION TYPE	MAX OBJECT SIZE	EXPOSURE/ PRINTING TIME	SPEED	MINIMAL FEATURE SIZE PRINTED	REPRESENTATIVE PRINTS	MATERIALS
**“Xolography”**[16]	Light-sheet	CW 375 nm, 70 mW, *SM<br><br>CW centre at 550 nm, *MM	Two Beams	4F	No	Amplitude	3 cm x 3 cm x 4 cm	1 – 8 min	55 m^3s^{-1}	~ 80 µm	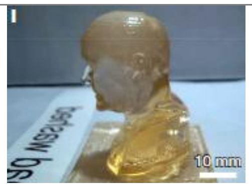 (Copyright Springer-Nature, reproduced with permission from the publisher)	Acrylates
**“Two-colour Light-Sheet”** [17]	Light-sheet	CW 440 nm, 6 W *MM<br><br>CW 660 nm, 3 W *SM	Two Beams	4F	No	Amplitude	0.5 µm x 0.5 µm x 2.2 µm	1 s	3.85 × 10^6 $µm^3\ s^{-1}$	~ 0.5 µm	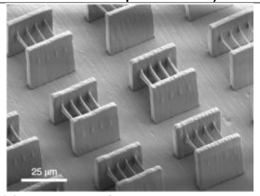 (Copyright Springer-Nature, reproduced with permission from the publisher)	Acrylates
**“Helical VAM”** [18]	Tomography	CW 405, 1.8 W *MM	One Beam	4F	No	Amplitude	3 cm x 3 cm x 5 cm	5 – 10 min	75 mm^3s^{-1}	~ 200 µm	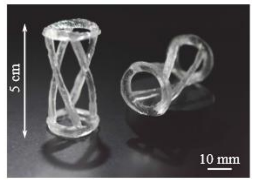 (Copyright Creative Commons CC BY 4.0)	Acrylates
**“High -resolution VAM”** [19]	Tomography	CW 405, 6.4 W *MM	One Beam	4F	No	Amplitude	1.7 cm x 1.7 cm x 2.3 cm	20 – 120 s	30 mm^3s^{-1}	~ 80 µm	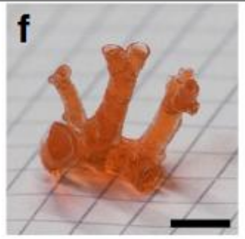 (Copyright Creative Commons CC BY)	Acrylates Hydrogels Ceramics Glasses Thiol-enes [20]
**“One-step VAM”** [21]	Interference	CW 532 nm, 135mW *SM	Three Beams	2F	Yes	Phase	6 mm x 6 mm x 6 mm	5 s	-	~ 100 µm - 200 µm	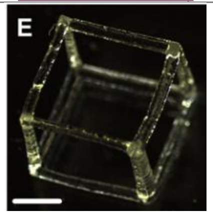 (Copyright AAAS, reproduced with permission from the publisher)	Acrylates
**“HoloVAM” (Current work)**	Interference + Tomography	CW 405 nm, 40mW *SM	One Beam	2F	Yes	Phase	2 mm x 2 mm x 2 mm	30 s – 160 s	-	~ 31 µm	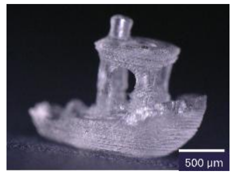	Acrylates Hydrogel

*MM: Multi-Mode spatial / SM: Single Mode spatial

Note: The cited articles provided all the data in the table.

Supplementary Note 11. Index Matching Bath: Magnification Analysis

To determine the possible magnification generated for a refractive index mismatch between the resin and the photoresin contained in the sample holder, we modeled the system using Zemax. We measure the refractive index of the vegetable oil and photoresin using a refractometer, which corresponds to $n_{resin} = 1.4833$ and $n_{oil} = 1.4763$ respectively. This results in a mismatch error of error of $n\Delta = 0.007$. We used ray tracing in the image plane (Fourier lens focus) and obtained spot diagrams (maps of the position of the ray intersecting the image plane) for three different points. In the first case, ray tracing in air, we could see that the reconstruction points are infinitesimally small, Supplementary Figure. 10.1 a. We also modeled the complete system, where the cylindrical glass resin container (diameter = 12 mm) is immersed on a glass cuvette with vegetable oil. The different surfaces, real dimensions and different refractive indices are considered. The spot diagrams obtained show a small magnification of the spots. However, the system does not introduce any significant magnification and provides magnification information of 1.004x, which is almost negligible.

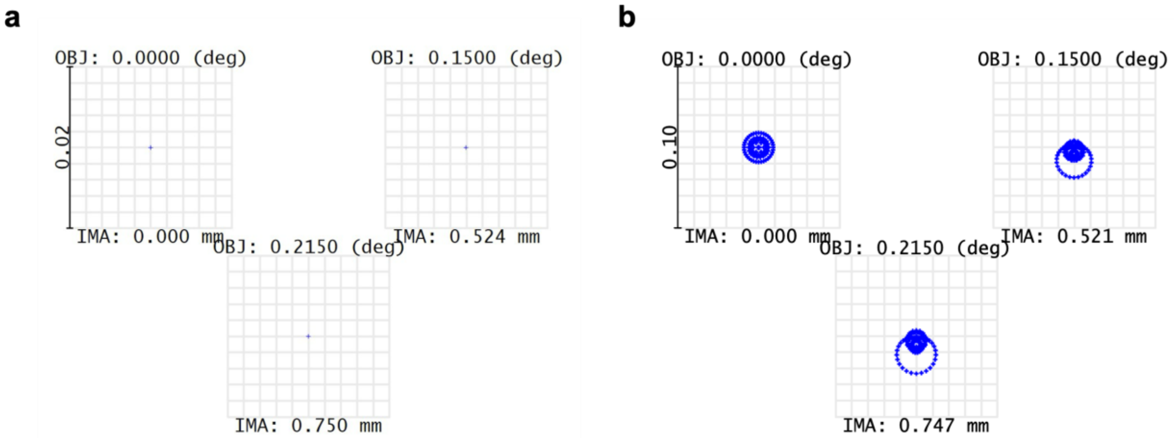

Supplementary Figure 10.1. Spot diagrams of the ray tracing simulations in Zemax**. a** Results of the spot diagram produced if the reconstruction image occurs in air. **b** Results of the spot diagram produced if the reconstruction image occurs after the ray propagated through all the surfaces and different mediums (air, oil, and resin). Scale bars (vertically located on the left side) are $0.02\ \mu m$ and $0.10\ \mu m$, respectively.

The simulated results are compared with the experimental results. A single holographic projection from the 3D model of a hole cylinder (Supplementary Figure 10.2.a) is calculated from a specific angle 0°. Images of the reconstructed intensity in the Fourier plane in the three different media (air, oil, and resin) are collected using the inspection camera 2. The size of the inner diameter was measured in the three different media. The size in air and in the resin is 715.36 μm and 719.20 μm respectively.The magnification is therefore 1.005x.

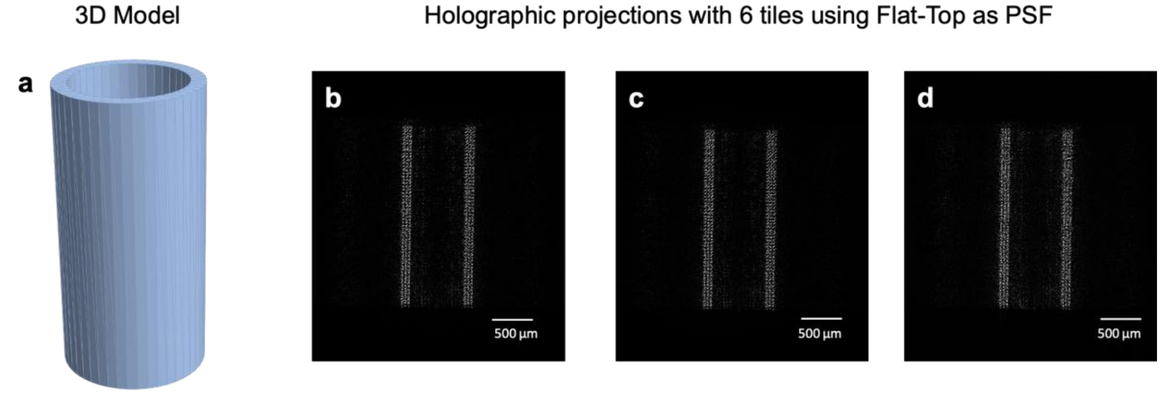

Supplementary Figure 10.2. a 3D model of a cylinder with a hole. **b-d** Reconstruction of the holographic projection corresponding to the 0° angle in air, oil, and resin respectively.

Supplementary Note 13. Stria reduction with PSF multiplexing

Statistical analysis. All values with physical units are shown in microns.

Supplementary Table 2. Results of ANOVA for differences across groups for stria Depth

Anova: Single Factor						
Stria Depth						
SUMMARY						
**Groups**	**Count**	**Sum**	**Average**	**Variance**		
L=1 vortex	14	76.85	5.49	3.90		
L=1 vortex + L=2 vortex	17	60.81	3.58	1.94		
Flat-top	16	101.74	6.36	6.88		
L=1 vortex + L=2 vortex + L=vortex + Flat-top	16	93.04	5.81	16.28		
ANOVA						

Source of Variation	SS	df	MS	F	P-value	F crit
Between Groups	73	3	24.362	3.349	0.025	2.761
Within Groups	429	59	7.275			
Total	502	62				

Supplementary Table 3. Results of ANOVA for differences across groups for stria Pitch

Anova: Single Factor						
Stria pitch						
SUMMARY						
**Groups**	**Count**	**Sum**	**Aver-age**	**Vari-ance**		
L=1 vortex	3	53.23	17.74	0.82		
L=1 vortex + L=2 vortex	3	30.93	10.31	0.43		
Flat-top	3	61.40	20.47	6.71		
L=1 vortex + L=2 vortex + L=vortex + Flat-top	3	54.65	18.22	6.77		
ANOVA						
**Source of Variation**	**SS**	**df**	**MS**	**F**	**P-value**	**F crit**
Between Groups	175	3	58.4	15.9	0.00099	4.1
Within Groups	29	8	3.7			
Total	205	11				

Supplementary Table 4. Results of Tukey HSD ad-hoc tests for differences across groups for stria pitch

**Pairwise_tukey HSD**						
**Pitch**						
**Multiple Comparison of Means - Tukey HSD, FWER=0.05**						
**Group 1**	**Group 2**	**Meandiff**	**p-adj**	**lower**	**upper**	**reject**
FT	L=1V + L=2V	-10.1568	0.001	-15.1743	-5.1392	True
FT	L=1-3V+FT	-2.2519	0.5124	-7.2695	2.7656	False
FT	L=1V	-2.724	0.3665	-7.7415	2.2936	False
L= 1V + L=2V	L=1-3V+FT	7.9048	0.0044	2.8873	12.9224	True
L=1V + L=2V	L=1V	7.4328	0.0063	2.4153	12.4503	True

L=1-3V+FT	L=1V	-0.472	0.9	-5.4896	4.5455	False

Supplementary Table 5. Results of Tukey HSD ad-hoc tests for differences across groups for stria depth

**Pairwise_tukey HSD**						
**Depth**						
**Multiple Comparison of Means - Tukey HSD, FWER=0.05**						
**Group 1**	**Group 2**	**Meandiff**	**p-adj**	**lower**	**upper**	**reject**
FT	L=1V + L=2V	-2.7813	0.0223	-5.2651	-0.2974	True
FT	L=1-3V+FT	-0.5438	0.9	-3.065	1.9774	False
FT	L=1V	-0.8693	0.7918	-3.479	1.7405	False
L= 1V + L=2V	L=1-3V+FT	2.2374	0.0918	-0.2464	4.7213	False
L=1V + L=2V	L=1V	1.912	0.2133	-0.6616	4.4857	False
L=1-3V+FT	L=1V	-0.3254	0.9	-2.9351	2.2843	False

Supplementary Note 14. Dose test

To quantify the printed object fidelity, multiple cylinders with a hole in the middle were printed, by varying the printing time. The cylinder height and perimeter of the inner hole where measured. From the results in the plots below, we can see that there can be small variations of features (height and perimeter) for printing time in the range of milliseconds. However, close to 100% fidelity could be obtained for a print time of 37 seconds.

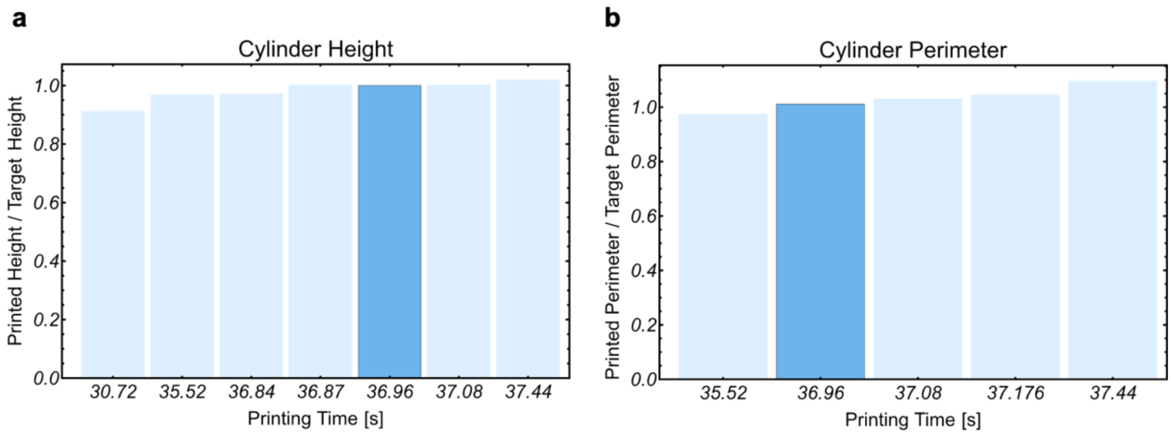

Supplementary Figure 11. Height **a** and perimeter **b** of a printed cylinder containing a hole in its middle as a function of printing time.

Supplementary Note 15. Print fidelity

Using microCT scans, we calculated the Jaccard similarity index, representing the intersection over union between the printed part and the microCT, where a Jaccard value of 1 indicates perfect agreement. Supplementary Figure 12 illustrates a typical 2D microCT slice alongside its corresponding 3D model slice for the four printed parts depicted in Fig. 7c. We observe a print fidelity of up to 0.86 for the Benchy boat produced with an acrylate-based resin using a Flat-top printer (Supplementary Figure 12b). The ~3% variation from other prints is attributed to the photochemistry of the photoresin, with TEMPO added to mitigate oxygen diffusion, enabling the printing of more intricate features.

Additionally, we assessed the fidelity of constructs printed in cell-laden hydrogel. Despite the potential deformation of soft hydrogel constructs, we used the Jaccard similarity index to evaluate fidelity through fluorescence microscopy images of stained cell nuclei. Supplementary Figure 12c presents a comparison of representative confocal microscopy cross-sections of the hydrogel construct (complex acinus) with the corresponding 3D model slices.

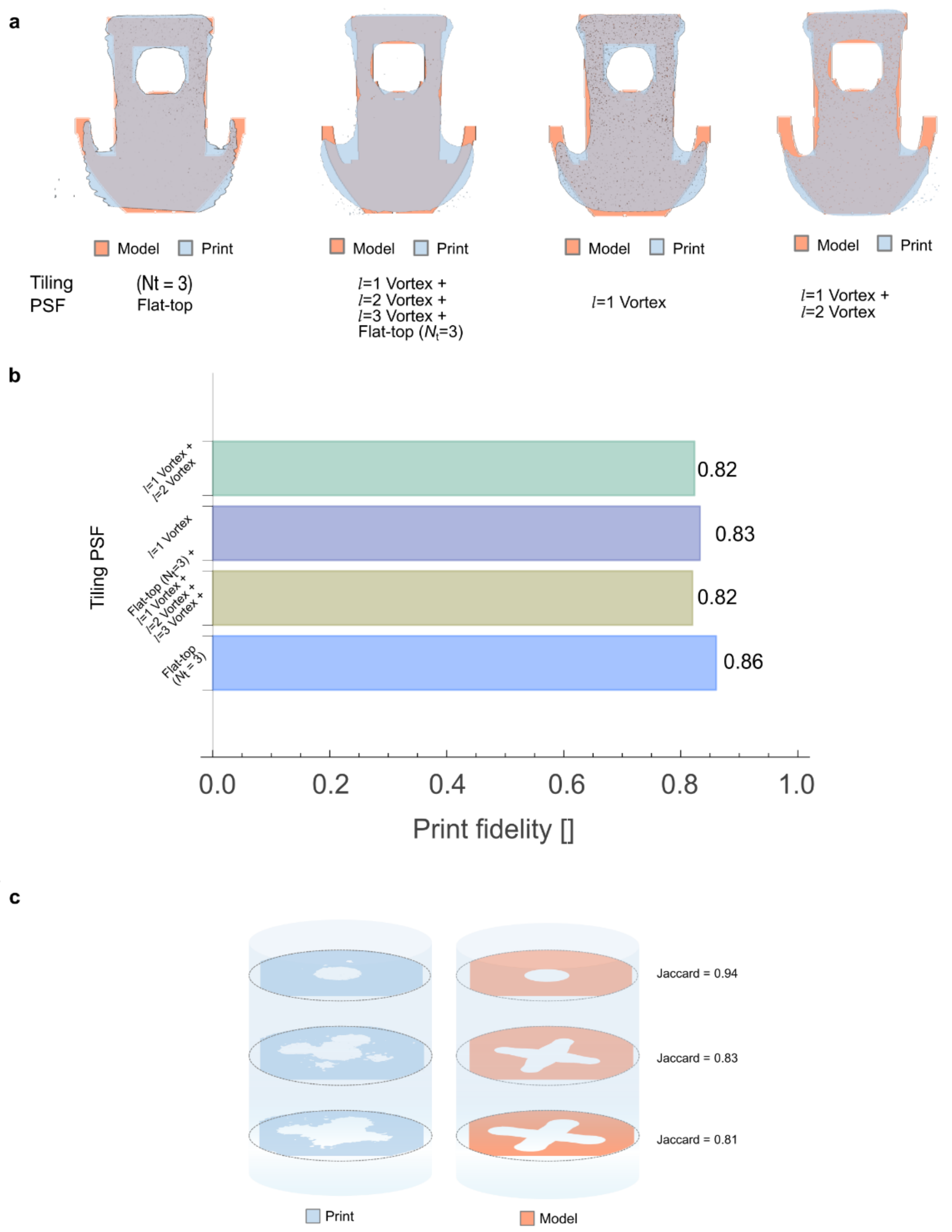

Supplementary Figure 12. a Comparison of a representative cross-section of the microCT image of a acrylate printed part (Benchy boats from Fig. 7c) with a 2D slice from the 3D model. **b** Measurements of print fidelity using the Jaccard similarity index as the intersection over the union of model and print, where Jaccard = 1 means perfect agreement. The measurements were performed for the 4 samples from Figure a. **c** Comparison of representative cross-sections of confocal microscope images of the hydrogel construct (complex acinus) with the 3D model**.**

Supplementary References

1. Wai-Hon Lee. Computer-generated hologram techniques and applications. *Progress in Optics,. E. Wolf Ed* **16**, (1978).
2. Gerchberg, R. W. & Saxton, W. O. A Practical Algorithm for the Determination of Phase from Image and Diﬀraction Plane Pictures. **35**, (1972).
3. Madsen, A. E. G., Eriksen, R. L. & Glückstad, J. Comparison of state-of-the-art Computer Generated Holography algorithms and a machine learning approach. *Optics Communications* **505**, 127590 (2022).
4. Glückstad, J. & Madsen, A. G. Comparing HoloTile with existing diffractive optics modalities. in *Complex Light and Optical Forces XVII* (eds. Andrews, D. L., Galvez, E. J. & Rubinsztein-Dunlop, H.) vol. 12436 124360P (SPIE, 2023).
5. Madsen, A. G. & Glückstad, J. HoloTile: Rapid and speckle-suppressed digital holography by matched sub-hologram tiling and point spread function shaping. *Optics Communications* **525**, 128876 (2022).
6. Glückstad, J. & Gejl Madsen, A. E. HoloTile light engine: new digital holographic modalities and applications. *Rep. Prog. Phys.* **87**, 034401 (2024).
7. Ren, Y., Lu, R. & Gong, L. Tailoring light with a digital micromirror device. *Annalen der Physik* **527**, 447–470 (2015).
8. Correa-Rojas, N. A., Gallego-Ruiz, R. D. & Álvarez-Castaño, M. I. Generation of linearly polarized modes using a digital micromirror device and phase optimization. *Computer Optics* **46**, (2022).
9. Glückstad, J. & Madsen, A. E. G. New analytical diffraction expressions for the Fresnel–Fraunhofer transition regime. *Optik* **285**, 170950 (2023).
10. Dickey, F.M., Dickey, F.M., Lizotte, T.E., Holswade, S.C., & Shealy, D.L. (Eds.). *Laser Beam Shaping Applications*. (CRC Press).
11. Dickey, F. M. Gaussian laser beam profile shaping. *Opt. Eng* **35**, 3285 (1996).
12. Goodman, Joseph W. *Introduction to Fourier Optics*. vol. 1 (Englewood, CO: Roberts \& Co. Publishers, 2005).

13. Curtis, J. E., Koss, B. A. & Grier, D. G. Dynamic holographic optical tweezers. *Optics Communications* **207**, 169–175 (2002).
14. Melville, H. *et al.* Optical trapping of three-dimensional structures using dynamic holograms. *Opt. Express* **11**, 3562 (2003).
15. Zhai, Z. *et al.* Parallel Bessel beam arrays generated by envelope phase holograms. *Optics and Lasers in Engineering* **161**, 107348 (2023).
16. Regehly, M. *et al.* Xolography for linear volumetric 3D printing. *Nature* **588**, 620–624 (2020).
17. Hahn, V. *et al.* Light-sheet 3D microprinting via two-colour two-step absorption. *Nat. Photon.* **16**, 784–791 (2022).
18. Boniface, A., Maître, F., Madrid-Wolff, J. & Moser, C. Volumetric Helical Additive Manufacturing. *Light: Advanced Manufacturing* **4**, (2023).
19. Loterie, D., Delrot, P. & Moser, C. High-resolution tomographic volumetric additive manufacturing. *Nat Commun* **11**, 852 (2020).
20. Madrid-Wolff, J. *et al.* A review of materials used in tomographic volumetric additive manufacturing. *MRS Communications* **13**, 764–785 (2023).
21. Shusteff, M. *et al.* One-step volumetric additive manufacturing of complex polymer structures. *Sci. Adv.* **3**, eaao5496 (2017).
