# Supplementary material for: Holographic tomographic volumetric additive manufacturing
Source: Nat Commun. 2025 Feb 11;16:1551. doi: 10.1038/s41467-025-56852-4 (PMC11814129; doi:10.1038/s41467-025-56852-4)
Supplement: Supplementary file 2 — Description of Additional Supplementary Files [file 41467_2025_56852_MOESM2_ESM.pdf]

Description of Additional Supplementary Files

Supplementary Data: Provides the raw data to create the graphs in Fig 3.

Supplementary Movie 1: Shows the printing process of a Benchy boat using holographic projection with 3 tiles.

Supplementary Movie 2: Shows the 3D reconstructions from the micro-CT scan of the printed sample in Fig. 6a.

Supplementary Movie 3: Shows the 2D slices from the micro-CT scan of the printed sample in Fig. 6a.

Supplementary Movie 4: Shows a scan of the 2D microscope images of the construct printed with the cell-laden hydrogel.
